# Development and validation of an AI-generated real-world object stimuli set

**DOI:** 10.3758/s13428-026-03021-0

**Published:** 2026-05-05

**Authors:** Gerard Campbell, Graeme Nicholls, Rebecca Hart, Richard J. Allen, Claudia C. von Bastian, Melanie R. Burke, Mario Parra Rodriguez, Louise A. Brown Nicholls

**Affiliations:** 1 Department of Psychological Sciences and Health, University of Strathclyde, 40 George St, Glasgow, G1 1QE UK; 2 Graeme Nicholls Architects, Glasgow, UK; 3 School of Psychology, University of Leeds, Leeds, UK; 4 Department of Psychology, University of Sheffield, Sheffield, UK

**Keywords:** Artificial intelligence, AI image generation, Real-world object stimuli, Validated stimuli, Visual Cognition

## Abstract

The availability of real-world object stimuli that meet researchers’ requirements is an ongoing challenge in visual cognition research. While numerous manually curated object stimulus sets exist, stimulus features such as size, color, and orientation tend to vary widely within a given set and may not be suitable for studies with specific requirements regarding these parameters. However, recent advances in artificial intelligence (AI) can facilitate the generation of highly realistic, custom-made stimuli. Building on these developments, the present study aimed to share a set of 200 AI-generated images of everyday objects for research use. The objects were oriented as though ‘placed’ on a flat surface, such that they could be naturally embedded in virtual scenes. Moreover, they were created in greyscale and suitable for rendering in different colors. Here, we report the method used to efficiently generate the stimuli, as well as the results from a validation study in which we assessed the nameability, perceived realism and familiarity of the stimuli in a sample of 45 younger (18–35) and 45 older (65–85) adults. As anticipated, the majority of the stimuli were rated highly across all three measures, and no significant age differences were observed. The results thus validated most of the stimuli for future research. The stimuli, each in seven colors, and the corresponding validation scores are openly available for future use. Low-level image statistics of mean brightness and contrast for each image are also included in the dataset.

## Introduction

Research on visual perception and cognition depends on the continued availability of realistic, novel, and diverse object stimuli. Traditionally, researchers have relied on manually curated object stimulus sets, which can be highly resource-intensive to create and may afford limited control over stimulus characteristics. However, recent advances in generative artificial intelligence (Gen-AI) can help address these challenges, allowing researchers to custom-create high-quality visual stimuli tailored to their specific requirements (Becker & Laycock, 2023; Luo & Toubia, 2024). Building on these developments, the present research aimed to validate and share an AI-generated set of real-world object stimuli for research use. The stimuli were created to be relatively uniform across key visual features (e.g., size, color), making them well-suited to controlled experiments. They are also available in seven different colors, and most are suitable for efficient batch processing if additional colors are required. Finally, objects were created to be oriented as though ‘placed’ on a flat surface, making them compatible with naturalistic designs in which objects are placed within virtual, everyday scenes. The stimuli set and corresponding validation scores are openly and freely available for use in future research (https://osf.io/tncdf/). Low-level image statistics (mean brightness and contrast), generated using MATLAB’s SHINE toolbox (Willenbockel et al., 2010), are also included within the dataset.

### Real-world visual object stimuli

Real-world visual object stimuli are essential tools for investigating visual perception and cognition (Brodeur et al., 2010). The continued availability of realistic, novel, and diverse object images is necessary to ensure that research findings are ecologically valid and generalizable across a broad range of stimuli (Becker & Laycock, 2023; Grootswagers & Robinson, 2021). In the cognitive neuroscience literature, manually curated object-image databases have become a highly popular resource for researchers. Numerous such databases exist (e.g., Adlington et al., 2009; Brady et al., 2008, 2013; Brodeur et al., 2010, 2014; Hebart et al., 2019; Moreno-Martínez & Montoro, 2012) and have been applied to a range of topics, including object recognition (Aminoff et al., 2022) and perception (Collins et al., 2019), semantic categorization (Hu & Jacobs, 2021), psycholinguistics (Lorenzoni et al., 2018), and machine learning (Bansal et al., 2023).

Despite their utility as scientific resources, creating these databases can be highly time- and resource-intensive, often involving physical collection and photography of thousands of objects (e.g., Brodeur et al., 2010) and/or extensive web searching and image editing (e.g., Brady et al., 2008). A further issue is that within a given image set, key visual attributes such as color, size, orientation, background, and verbal content (e.g., labels, digits) tend to vary across images and may not be suitable for experiments with specific requirements for these parameters. For instance, in many existing sets, objects are not consistently oriented such that they could appear naturally on a flat surface. Therefore, existing images are mainly useful for studies in which objects are presented without background scene context and are less suitable for more ecologically valid paradigms in which objects are presented within virtual, everyday scenes, such as around the home. Additionally, most existing object–image databases provide stimuli in only one color. However, visual cognition researchers often require the same object in multiple colors. Notably, Brady and colleagues (2013) have developed a set of 540 object stimuli designed to be color-manipulable, that is, the object images generally have a single color and can be efficiently batch-processed to generate other colors. However, this stimulus set does not include any validation measures, such as stimulus nameability and familiarity.

Overall, while some of the above issues can be addressed through manual editing and/or stimulus selection, this can be time-consuming and inefficient, depending on researchers’ needs and the amount of editing required. To address limitations in existing object databases, we used AI to efficiently generate a set of real-world object stimuli in greyscale and in six other colors.

### Generative artificial intelligence as a tool for visual image creation

Recent advances in gen-AI offer promising opportunities for the custom creation of novel visual stimuli, including real-world object images. Recently developed text-to-image models, such as DALL-E 3 (OpenAI, 2023) and Stable Diffusion 3.5 (Stability AI, 2024), allow for instantly generating and modifying highly realistic images using text prompts. These programs are based on deep learning algorithms that have been trained on large datasets of images and text, allowing them to interpret user text input and generate images with high levels of detail and accuracy. Another prominent approach, known as generative adversarial networks (GANs), uses a machine learning algorithm to generate novel sets of visual outputs (e.g., images, videos) based on training data (Goodfellow et al., 2014). Importantly, research has shown that humans are poor at discriminating Gen-AI images and real images (Bray et al., 2023; Huang et al., 2024; Lu et al., 2023), demonstrating that images produced by the technology can be perceived as highly realistic.

In line with these developments, there is a growing literature exploring the utility of AI for generating realistic visual stimuli for research purposes. For instance, Wei et al., (2024a, 2024b) have recently developed the AI model CoCoG (Concept-based Controllable Generation) capable of generating scenes in which the visual prominence of a given concept can be systematically varied while other image features are preserved. Wei et al. (2024a) proposed that the model can be used to create well-controlled stimuli for tasks on conceptual representation and decision-making, such as similarity judgment tasks. In face processing and recognition research, several studies have used AI-generated photos (Luo & Toubia, 2024) or videos (Eberl et al., 2022; Haut et al., 2021) to manipulate features of interest (e.g., facial femininity, race) independently of other attributes (e.g., attractiveness, expression). As noted by Luo et al. (2024), the use of AI to systematically isolate and vary specific facial features represents a significant methodological breakthrough, which has been extremely difficult to achieve with manually curated stimulus sets.

Of particular relevance to the present work, one study has used AI to generate a set of novel object-like stimuli (Cooper et al., 2023). The authors used GANs that were trained on images of real objects to produce a set of 400 synthetic object-like stimuli that appear realistic but do not depict actual objects. In a subsequent validation study, the authors found that AI-generated objects were perceived as less familiar than a comparable set of manually curated real-world object images, though they were rated as similarly engaging. The authors highlighted that the stimuli can be used to facilitate research on how the brain processes perceptually novel objects. This research further demonstrates the utility of AI in generating novel, object-like stimuli for use in research.

### The present study: AI-generated real-world object stimuli set

Building on these developments, the aim of the present research was to validate and share an AI-generated real-world object stimuli set for use in future research. The stimuli were developed for a series of visual working memory experiments in which younger and older adults would be asked to remember household objects placed within realistic virtual scenes. The stimuli were created to be free from readable text (e.g., letters, numbers), suitable for color rendering, and to be relatively similar across visual attributes such as size and orientation. Specifically, objects were oriented so that they could be perceived as sitting on a flat surface within the scene. We aimed to produce objects that would be similarly familiar across younger and older adults. Importantly, we originally explored existing object stimuli sets for potential use in our experiments (e.g., Brady et al., 2008; Brodeur et al., 2010). However, the images tended to be unsuitable in some of these respects (e.g., too variable in size in the real world) and/or would require significant editing (e.g., rotation, tilt) to meet our specific requirements.

In the present work, we report the image generation method we used to create the stimuli, as well as a subsequent study in which we validated the stimuli for nameability, perceived realism, and familiarity in a sample of younger and older adults. The final set of 200 objects and their corresponding validation scores are openly and freely available for use in future research (https://osf.io/tncdf/). In addition, the stimuli are each available in seven different colors, with most being suitable for efficient batch-processing in other colors, as required. Finally, low-level image statistics (mean brightness and contrast) for each image, generated using MATLAB’s SHINE toolbox (Willenbockel et al., 2010), are also included within the dataset.

## Image generation method

### Software

The stimuli were generated using Adobe Firefly (Adobe, 2024), an advanced AI tool that generates images based on user text prompts. Most images were created using the Image 2 model but a proportion was created using the more advanced Image 3 model upon its release in beta format. In addition, Adobe Photoshop image editing software was used for postprocessing of the stimuli following image generation (see section ‘Image postprocessing’ for details).

### Stimuli specification

The stimuli were created for tasks in which participants were asked to remember everyday objects placed within virtual household scenes (specifically, a kitchen, a living room, and a home office). A list of over 200 objects was initially created by the researchers. Broadly, this consisted of relatively small, household items that could fit on a household surface (e.g., approximately sized between a set of keys and a table lamp). In addition, the specification for the images were: (1) each image should appear reasonably realistic and contain either one object only or two objects as an associated pair (e.g., candle and holder); (2) objects should be face- or side-on without angle or tilt, such that they could appear naturally placed on a surface; (3) all objects should initially be generated in a neutral color (i.e., silver; r = 192, g = 192, b = 192), with a transparent background^1^; (4) there should be no textual information (e.g., letters, digits, brand names); (5) images should be generated in a high resolution (1000 × 1000 px) to maximize future use of the set; (6) other visual features such as brightness, contrast and saturation should be relatively consistent across images; and (7) the objects should be recognizable across younger and older adults (i.e., no particularly modern or outdated objects).

### Image generation

Based on the specifications, we developed a set of descriptive keywords and phrases that were used as text prompts to generate the images. The prompts were input to Adobe Firefly for each object, one at a time. Each prompt generated four image options, and the one deemed best, according to the criteria above, was selected for potential inclusion in the stimulus set. After a process of trial and refinement, specific program settings were found to give optimal results. These settings are available in the online supplementary materials (https://osf.io/8wbtk). Regarding prompt format, the following was found to produce the most consistent results and was used for the majority of the objects:

“*Typical [item name]. Light grey color. Front (or side) view on tabletop. Plain white background. Item only*”.

In some instances, minor refinements to the wording or additional detail were needed to achieve satisfactory results. Prompts typically required fine-tuning when objects appeared unnaturally balanced or when a different viewing angle was needed to make them more recognizable. For example, objects with handles (e.g., scissors, hairbrush) were occasionally generated standing upright rather than resting flat as they naturally would on a surface. In such cases, amending the prompt to specify that the item should be lying flat on the table was sufficient to generate an appropriate image.

**Fig. 1:**
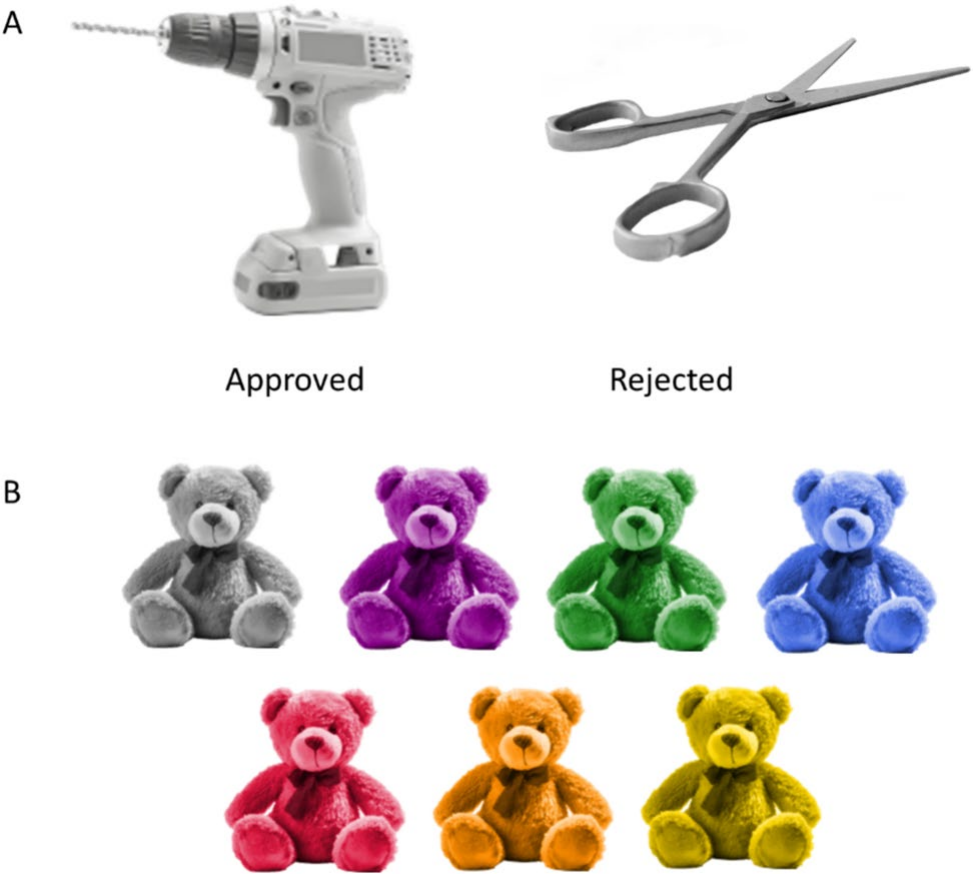
Example stimuli generated by Adobe Firefly. *Note.* **A** Examples of images that were approved and rejected during the initial review process, based on the authors’ perceived image accuracy. The scissors were rejected due to the handles appearing misshapen/incomplete. **B** Example AI-generated stimulus (teddy bear) in neutral (‘silver’) color and the six different color versions (‘dark magenta’, ‘forest green’, ‘royal blue’, ‘crimson’, ‘dark orange’, ‘gold’)

Overall, the resulting images were of high quality. However, in some cases the objects were found to be obviously less realistic and/or did not accurately resemble the intended object, even after refining the initial prompt. For example, one consistent limitation with the earlier version of Adobe Firefly was its difficulty in accurately generating images of electrical products. However, the updated version was able to generate much more accurate and realistic images even for this object type. The final stimuli set, consisting of 200 images, was agreed upon by the authors following an ongoing, iterative review and feedback process to select the images with the highest perceived accuracy and suitability with respect to the predefined image specifications. Examples of approved and rejected images from this initial review process are shown in Fig. 1A.

### Image post-processing

The set of 200 images were then processed in Adobe Photoshop image editing software. To isolate the object, the background was removed using the ‘remove background’ smart tool, with additional manual editing where required.^2^ Then, the image was desaturated to create a neutral base image (i.e., silver; r = 192, g = 192, b = 192) using an action (i.e., a short, premade script in which users can record a series of steps that can be applied to multiple images at once). Next, six color versions of the images were produced in ‘crimson’ (r = 220, g = 20, b = 60), ‘dark magenta’ (r = 139, g = 0, b = 139), ‘dark orange’ (r = 255, g = 140, b = 0), ‘forest green’ (r = 34, g = 139, b = 34), ‘gold’ (r = 255, g = 215, b = 0), and ‘royal blue’ (r = 65, g = 105, b = 225). Following this, Adobe Photoshop’s editing tools were used to adjust elements such as brightness, contrast and saturation so that these were perceived to be relatively uniform across the stimuli. Finally, seven versions of each stimulus (i.e., the silver and the six other color versions) were saved as transparent PNG image files. An example image (teddy bear) in all seven colors is shown in Fig. 1B.

## Validation of the AI-generated stimuli set

We conducted a study to validate the stimuli on key characteristics and to establish the suitability of each stimulus for use in our own and others’ future research. Specifically, this study aimed to validate the stimuli in terms of nameability, perceived realism, and familiarity. For our own research, the stimuli needed to comprise realistic, nameable objects. In addition, we assessed perceived familiarity as this is commonly considered in the field (Brodeur et al., 2014; Ngo & Lloyd, 2018; Sunday et al., 2022) and, therefore, is likely a useful measure for other researchers. Finally, because we intended to use the stimuli with both younger and older adult participants, we analyzed these variables by age group to assess any potential age-related effects.

### Methods

#### Participants

The study was ethically approved by the Department of Psychological Sciences and Health Ethics Committee at the University of Strathclyde and administered online using the recruitment platform Prolific (Prolific, 2024) and the stimulus presentation software Gorilla (Anwyl-Irvine et al., 2020). The participants were 45 younger and 45 older adults, all based in the UK (see Table 1 for demographics, both overall and by age group). The sample size was based on previous stimulus validation studies (Brodeur et al., 2010, 2014; Rosedahl & Ashby, 2018). Participants met the following inclusion criteria via self-report: aged between 18 and 35 or 65 and 85, fluent in English, with normal or corrected-to-normal vision, no current diagnosis of dyslexia or a mental health condition, and, for older adults only, no diagnosis of mild cognitive impairment or dementia. Participants were compensated with £5 for their time. No participants were excluded from the final sample.

**Table 1:** Participants’ demographic data

Variable	Young (18–35)	Older (65–82)	Overall
*N* (% of sample)	45 (50%)	45 (50%)	90 (100%)
Age (*M* ± *SD*)	27.47 (5.20)	68.80 (3.78)	48.13 (21.27)
Gender			
Male	14 (31.1%)	21 (46.7%)	35 (38.9%)
Female	31 (68.9%)	24 (53.3%)	55 (61.1%)
Non-Binary/Other	-	-	-
Prefer not to say	-	-	-
English first language			
Yes	40 (88.9%)	44 (97.8%)	84 (93.3%)
No	4 (8.9%)	1 (2.2%)	5 (5.6%)
Prefer not to say	1(2.2%)	-	1 (1.1%)
Ethnicity			
White	30 (66.7%)	43 (95.6%)	73 (81.1%)
Asian/Asian Scottish or British	6 (13.3%)	1 (2.2%)	7 (7.8%)
Black/Black Scottish or British, African or Caribbean	7 (15.6%)	1 (2.2%)	8 (8.9%)
Mixed/multiple ethnic groups	1 (2.2%)	-	1 (1.1%)
Other (please specify)	1 (2.2%)	-	1 (1.1%)
Prefer not to say	-	-	-
Education (yrs; *M* ± *SD*)	15.76 (3.36)	14.71 (3.29)	15.23 (3.35)
Highest level of education completed			
No schooling completed	-	-	-
Primary	-	-	-
Secondary/High School	5 (11.1%)	10 (22.2%)	15 (16.7%)
Further Education/College	11 (24.4%)	10 (22.2%)	21 (23.3%)
Undergraduate	19 (42.2%)	12 (26.7%)	31 (34.4%)
Postgraduate	9 (20%)	11 (24.4%)	20 (22.2%)
Doctorate	-	2 (4.4%)	2 (2.2%)
Prefer not to say	1 (2.2%)	-	1 (1.1%)
Current employment status			
Full-time employment	28 (62.2%)	1 (2.2%)	29 (32.2%)
Part-time employment	4 (8.9%)	4 (8.9%)	8 (8.9%)
Unemployed	5 (11.1%)	2 (4.4%)	7 (7.8%)
Self employed	2 (4.4%)	1 (2.2%)	3 (3.3%)
Student	4 (8.9%)	-	4 (4.4%)
Retired	-	37 (82.2%)	37 (41.1%)
Prefer not to say	2 (4.4%)	-	2 (2.2%)

Data represent number and percentage of sample unless otherwise stated. Percentages are calculated within groups. Due to rounding, percentages do not always total 100

#### Materials

The stimuli for the study comprised all 200 object images from the stimuli set in silver color. The images were presented in the top center of the screen with a position of *x* = 0, *y* = – 5 and a size of 5 × 5 (*x*, *y*) using Gorilla’s coordinate system. Gorilla’s device requirements were set to allow study completion via a computer or tablet, but not via mobile phones.

#### Procedure

Participants first carried out the required screening questionnaire on Prolific. Participants who passed the screening were then redirected to Gorilla, where they underwent an additional screening, gave informed consent, completed a brief demographics questionnaire, and then read the task instructions. Participants were informed that they would be shown one object at a time and asked to type its name (or a description) and rate it on realism and familiarity. Participants were informed that the purpose of the study was to assess the validity of the images for future research and that they should aim to respond as honestly as possible.

**Fig. 2:**
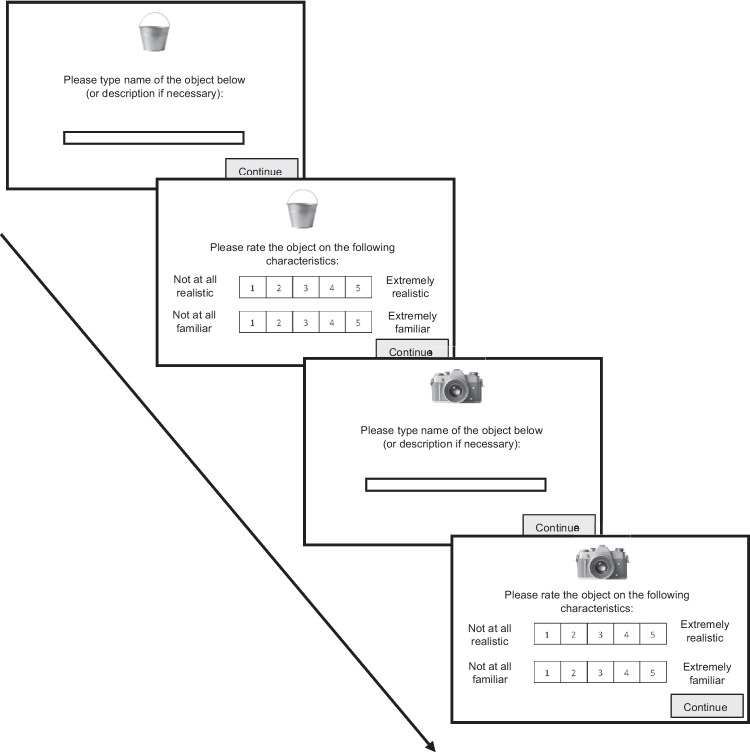
Illustration of the experimental paradigm for two example stimuli. *Note.* Participants were presented with a random sequence of 80 objects from the stimulus set. For each object, participants first typed its name or description, then rated it for realism and familiarity. Task content is for illustrative purposes only and is not drawn to scale

Figure 2 illustrates the study procedure. Participants were asked to provide names that are as brief and unambiguous as possible (Brodeur et al., 2010; Snodgrass & Vanderwart, 1980). If participants were unable to name a given object, they could provide a brief description instead. For images that contained more than one object (e.g., a cigarette in an ashtray), participants were asked to name/describe the largest or most prominent object. If participants were unable to name or describe the object, they were asked to indicate this clearly in their response (e.g., “I do not recognize this object”). Participants were required to type a response before being able to progress in the task by clicking the ‘continue’ button after typing their response. Finally, participants were asked to rate the objects in terms of realism and familiarity based on the following definitions: “Realism refers to the extent to which the image resembles the object as it could be perceived in real life. Familiarity refers to how well you recognize the object or have knowledge of it”.

After the instructions, participants were presented with a sequence of 80 randomly selected images from the stimulus set. Across all participants, each image was presented an average of 36 times (*SD* = 4.83, min = 23, max = 49). All images were presented at the top and center of the screen in neutral silver. Participants first saw the image with the following message underneath: “Please type the name of the object below (or description if necessary):”. A textbox was provided below for participants to type their response (Fig. 2). After pressing ‘continue’, on the next screen participants were again presented with the same object and asked to rate it in terms of realism and familiarity using the five-point Likert scales presented below the image: 1 = *not at all realistic/familiar*, 5 = *extremely realistic/familiar*. This process was repeated for all 80 images, and participants were given an optional break after 40 images. There was no time limit for participants to make their responses. The average time to complete the task was 22 min (*SD* = 8.64).

#### Nameability coding procedure

To assess object nameability, participants’ typed responses were coded across two separate categories: (1) ‘match type’ and (2) ‘response type’. ‘Match type’ referred to whether a given participant’s name or description matched the intended object. There were only two possible codes in this category: (1) ‘match’ and (2) ‘non-match’. A flexible approach was used to assign matches, such that if the participant’s name or description reasonably approximated the intended object, then it was coded as a match (e.g., “food processor” and “blender” were acceptable as matches for the ‘smoothie maker’). In addition, responses that contained minor spelling mistakes were also coded as ‘match’, provided the intended response was clear.

There were four possible codes for ‘response type’: (1) ‘named’ – the participant provided a name for the object (generally 1–3 words), or a description which clearly contained the intended object’s name; (2) ‘described’ – the participant provided a description which did not contain the object’s name; (3) ‘object unknown’ – the participant indicated that they did not know/recognize the object; and (4) invalid response – no clear response was given. Example responses and codes assigned are in the online supplementary materials (https://osf.io/s8bqu).

One researcher (GC) coded all responses across the 200 objects, and a second researcher (RH) coded all responses for 20% of the objects (i.e., 40) as a reliability check. Inter-coder reliability, assessed using Krippendorff’s alpha, was good (*α* =.84; Krippendorff, 2019). Thus, codes assigned by GC were used in the analysis. Once all objects had been coded for both match and response type, combined codes collapsing across these two categories were then created (i.e., match-named, match-described, non-match-named, etc.). To assess nameability, the main measure used was the proportion of responses coded as matched and named for each object, with a higher proportion indicating higher nameability.

### Results

The stimuli set, raw data, and a CSV file containing the data related to each stimulus are freely accessible on the Open Science Framework (https://osf.io/tncdf/). The data include the nameability, realism, and familiarity scores for each object, both across all participants and by age group. Here, we report the overall mean scores for these measures and test for any age-related differences.

Before conducting the analysis, the data were checked for outliers using boxplots. One participant’s nameability score was identified as an extreme outlier (i.e., scoring above or below the upper or lower quartile, plus or minus 3 × the interquartile range) and therefore was removed from that analysis. Descriptive statistics of the remaining sample’s data (overall and by age group) are shown in Table 2.

**Table 2:** Descriptive statistics for nameability, realism, and familiarity (overall and by age group)

	Young	Older	Overall
Nameability			
Proportion correct (match-named; ± *SD*)	.94 (.04)	.95 (.03)	.94 (.04)
Min	.81	.84	.81
Max	.99	1	1
Realism			
Mean (± *SD*)	4.38 (.57)	4.49 (.43)	4.43 (.50)
Min Max	2.36 5	3.21 5	2.36 5
Familiarity			
Mean (± *SD*)	4.35 (.57)	4.48 (.41)	4.42 (.50)
Min Max	2.55 5	3.31 5	2.55 5

To test for effects of age group across the three measures, we used independent *t* tests supplemented with Bayes factors. *BF*_10_ indicates the strength of the evidence in favor of the alternative hypothesis. *BF*_10_ < 1 indicates support for the null hypothesis. *BF*_10_ = 1–3 is considered as indicating ambiguous evidence for the alternative hypothesis, *BF*_10_ = 3–10 as substantial evidence, and *BF*_10_ > 10 as strong evidence (Keysers et al., 2020; Wetzels et al., 2011).

Regarding nameability, no significant difference was found between the younger and older adults, *t*(87) = 0.38, *p* =.70, *BF*_10_ = 0.24. This was also the case for realism, *t*(88) = 1.05, *p* =.29, *BF*_10_ = 0.36, and familiarity, *t*(88) = 1.29, *p* =.20, *BF*_10_ = 0.46. Thus, age group did not reliably affect the overall nameability, perceived realism, or familiarity of the stimuli set.

## Discussion

The present research aimed to validate and share an AI-generated real-world object stimuli set for use in visual cognition research. The stimuli were created such that they could be easily embedded in real-world scenes, and their colors could be readily changed according to the researchers’ requirements. Furthermore, the stimuli were validated in a sample of younger and older adults, highlighting their suitability for use within both age groups.

### Stimuli validation

The results of the validation study highlight the suitability of most of the stimuli for use in future research. Specifically, the overall high nameability scores show that the majority of stimuli represent nameable, recognizable objects, as intended. They are thus generally appropriate for research requiring objects that are clearly identifiable and perceptually unambiguous, such as studies on real-world object recognition, memory, or semantic categorization. Furthermore, the high realism scores show that the stimuli were generally perceived as representative of real-world objects. This highlights the ecological validity of the stimuli, and their suitability for studies requiring naturalistic object images. Finally, with regards to familiarity, ratings on this measure were also generally high, and the overall mean familiarity rating (4.42, *SD* =.50) was similar to those observed in other stimuli sets with a high proportion of familiar everyday objects. For example, in their validation of the Bank of Standardized Stimuli (BOSS), Brodeur and colleagues reported a mean score of 4.0 (*SD* =.4) for the first set of object images (Brodeur et al., 2010), and a mean score of 4.16 (*SD* =.5) for the subsequent set (Brodeur et al., 2014).

It is important to highlight that, while overall scores across the three measures were high, there is of course variation across items in terms of their individual ratings. Regarding nameability, for instance, the majority of objects have a nameability score of 80% or higher, but there is a small subset of objects (*n* = 12) for which the nameability score ranges between 30 and 73%. Researchers can thus use our by-item table (https://osf.io/tncdf/) to select stimuli according to their specific requirements. In our own research, for instance, objects with a nameability score less than 90% were either reserved for practice trials or excluded. However, future research may specifically seek variability in this or the other measures, depending on the specific research questions, therefore all 200 stimuli were retained in the openly available set.

Finally, the study revealed no significant age-related differences for any of the three main measures, suggesting that the stimuli were perceived similarly across age groups. These factors should therefore not confound research using the stimuli across younger and older adults, particularly when using a random subset of the stimuli. Again, however, there is variation across individual items in terms of how they were perceived across age groups. Importantly, our supplementary by-item table also includes a breakdown of item scores by age group, allowing researchers to select stimuli that are best matched across age groups, if required.

### Utility of Gen-AI for creating real-world object stimuli

Secondary to our central aim of developing the stimulus set, we highlight the utility of AI for efficiently generating object stimuli. While we aimed to develop relatively small household objects, a similar approach could be used to generate larger objects (e.g., furniture, vehicles), natural objects (e.g., plants) and less common objects (e.g., weapons, historical artifacts; Brodeur et al., 2014). In addition, we focused on developing greyscale objects suitable for rendering in different colors, given the lack of stimulus sets that meet this requirement. However, studies requiring more naturalistic stimuli could use AI to generate objects in their real-world color. Finally, as with most existing stimulus sets, our object images do not contain any background scene/context. However, numerous AI models now exist that can generate highly realistic scenes with embedded objects (Cheng et al., 2022; Wei et al., 2024a, 2024b), thus offering valuable opportunities for creating scene-based stimuli.

It is important to emphasize that, although AI can be a highly useful tool for efficient stimuli creation, AI-generated outputs still require a significant degree of consideration, refinement, and human validation. This may include modifying text prompts through trial-and-error, manual quality control checks to ensure that stimuli meet requirements, and/or post-image processing to adjust stimuli features. An additional consideration is that the quality of the results depends on the AI software used. A wide range of text-to-image generators exist, all of which vary in terms of image quality, user control, and accessibility (Goring et al., 2023; Muthaiah et al., 2024). While the software used here (Adobe Firefly; Adobe, 2024) requires a paid subscription, there are several free and open-source tools that offer both high realism and flexibility (e.g., Stable Diffusion, Dall-E; see Muthaiah et al., 2024 for a review). A final point is that newer versions of specific models may significantly enhance results. For instance, we observed that the later model of Adobe Firefly (Image 3 Model; Adobe, 2024) was considerably better at generating accurate, realistic images of electrical products than the previous version.

### Ethical considerations

While AI has the potential to greatly enhance the generation of object stimuli, it is important to ensure it is used ethically and with awareness of the technology’s risks and limitations. First, in accordance with international guidelines (e.g., European Commission, 2025) and general open science principles, researchers should be fully transparent on the use of AI to generate stimuli. This includes providing the name and version of the AI tool, details of the inputs (e.g., text prompts, training data) and any additional settings/parameters used. Second, it is important to recognize that AI models can produce inaccurate, misleading, and/or offensive content. In particular, due to significantly biased training data, AI models can produce images of people and objects that reflect widely held ethnical and social stereotypes (Bianchi et al., 2023). While it is currently challenging to manually overcome these biases, researchers should nonetheless take potential biases into account and minimize their impact where possible (Bianchi et al., 2023; European Commission, 2025). Finally, given that Gen-AI consumes significant energy resources and can have negative environmental impacts (de Vries, 2023), researchers should avoid using AI to generate stimuli unnecessarily, that is, in the absence of a clear research purpose, and limit its use to producing only what is required.

### Limitations

Regarding limitations of the present work, the validation study sample only included English-speaking participants located in the UK, and observed scores for nameability, realism and familiarity may not generalize to other populations. Future research could validate the stimuli in other populations, given that cross-cultural and cross-linguistic effects have been observed for manually curated object stimuli, particularly regarding familiarity (Santos et al., 2019). Furthermore, a limitation of the stimuli set is that only the greyscale images were included in the validation study, given that validating all 1400 object–color combinations would have been unfeasible within our work. Thus, while the color versions of the stimuli are available for future use, our current validation scores for the images only apply to the greyscale images.

### Summary

The current work presents an openly available AI-generated real-world object stimuli set with corresponding validation measures of stimuli nameability, realism, and familiarity. A salient advantage of the stimuli is their relative uniformity across key visual features (e.g., size, color, orientation), making them well suited to controlled experimental approaches. They are also available in seven different colors, and most are suitable for efficient batch processing if additional colors are required. Furthermore, all objects are oriented as though ‘placed’ on a flat surface, making them compatible with naturalistic designs in which objects are placed naturally within virtual scenes (e.g., Evans & Wolfe, 2022). Finally, the stimuli have been validated in both younger and older adults, demonstrating their suitability for use with both age groups. The stimuli and corresponding validation data are openly available for use in future research (https://osf.io/tncdf/). In addition, the present work provides a valuable resource for other researchers seeking to generate their own stimuli using gen-AI.

## Data Availability

The data and stimuli from the validation study are publicly available through the Open Science Framework (https://osf.io/tncdf/). The validation study task is publicly available on Gorilla’s Open Materials repository (https://app.gorilla.sc/openmaterials/1060453).

## Footnotes

1 A small number of items were not suitable for complete rendering in a single color. In such cases, silver was used in combination with a second color (e.g., brown egg in silver egg cup).

2 Note that a small number of objects required manual editing to successfully remove the background. This included objects with fine detail, or lighter areas that could be confused with the white background.

## Code availability

The code to reproduce the analyses is available on the Open Science Framework (https://osf.io/tncdf/).
